# Einstein, von Frisch and the honeybee: a historical letter comes to light

**DOI:** 10.1007/s00359-021-01490-6

**Published:** 2021-05-10

**Authors:** Adrian G. Dyer, Andrew D. Greentree, Jair E. Garcia, Elinya L. Dyer, Scarlett R. Howard, Friedrich G. Barth

**Affiliations:** 1 School of Media and Communication, RMIT University, Melbourne, VIC 3001 Australia; 2 Department of Physiology, Monash University, Clayton, VIC 3800 Australia; 3 ARC Centre of Excellence for Nanoscale BioPhotonics, School of Science, RMIT University, Melbourne, VIC 3001 Australia; 4 Department of Computer Science and Software Engineering, Swinburne University of Technology, Hawthorn, VIC 3122 Australia; 5 Centre for Integrative Ecology, School of Life and Environmental Sciences, Deakin University, Burwood, VIC 3217 Australia; 6 Department of Neurosciences and Developmental Biology, Faculty of Life Sciences, University of Vienna, Althanstr.14, 1090 Vienna, Austria

**Keywords:** Albert Einstein, Karl von Frisch, Insect, Skylight polarization pattern, Long distance navigation

## Abstract

The work of the Nobel Laureate *Karl von Frisch*, the founder of this journal, was seminal in many ways. He established the honeybee as a key animal model for experimental behavioural studies on sensory perception, learning and memory, and first correctly interpreted its famous dance communication. Here, we report on a previously unknown letter by the Physicist and Nobel Laureate *Albert Einstein* that was written in October 1949. It briefly addresses the work of von Frisch and also queries how understanding animal perception and navigation may lead to innovations in physics. We discuss records proving that Einstein and von Frisch met in April 1949 when von Frisch visited the USA to present a lecture on bees at Princeton University. In the historical context of Einstein’s theories and thought experiments, we discuss some more recent discoveries of animal sensory capabilities alien to us humans and potentially valuable for bio-inspired design improvements. We also address the orientation of animals like migratory birds mentioned by Einstein 70 years ago, which pushes the boundaries of our understanding nature, both its biology and physics.

## Innovation by critical thinkers: Einstein and von Frisch

As science and technology advance, so too does the specialization at their frontiers. This specialization can have the unfortunate consequence of isolating thinkers and breaking apart research disciplines. Reacting against this development is transdisciplinary fields bringing together experts from different disciplines to solve common problems. Such barriers between disciplines often did not exist until fairly recently, and where they did exist, they were porous to the great minds of their time.

**Fig. 1:**
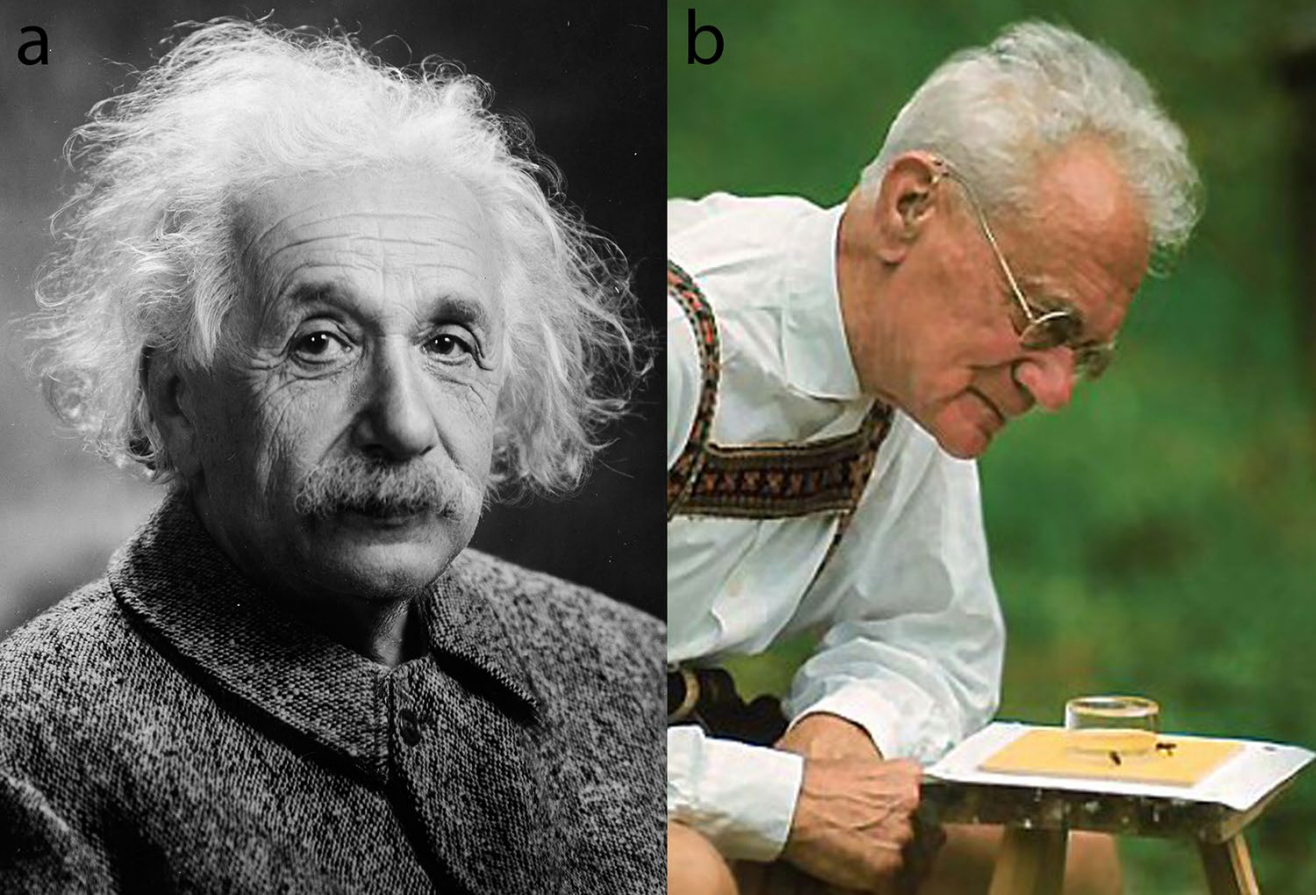
Key thinkers about physics and nature. **a** Professor Albert Einstein in 1947**.** From Wikimedia Commons, the free media repository. **b** Professor Karl von Frisch observing bees. From Wikipedia By Source (WP:NFCC#4), Fair use, https://en.wikipedia.org/w/index.php?curid=50796040

*Albert Einstein* (Fig. 1a) is widely recognized as one of the greatest thinkers of the twentieth century. His imagination and insight still inspire us today. Einstein’s work on quantum mechanics directly led to the transistor revolution and the information age, while his theory of general relativity governs the large-scale structure of the universe and provides the necessary corrections for location determination by the Global Positioning System (GPS). Nevertheless, it might be easy to pigeonhole Einstein as a mathematician and theoretical physicist, concerning himself solely with an abstract world of numbers and equations. That Einstein was also concerned with practicalities and had a broad interest in research and humanity is well discussed in Galison’s book “Einstein’s clocks, Poincare’s maps: empires of time” (2003).

*Karl von Frisch* (Fig. 1b) founded the present journal together with *Alfred Kühn* in 1924. By employing innovative experimental techniques to understand how bee behaviour worked (von Frisch 1915, 1923, 1949, 1965, 1967, 1973, 1974), von Frisch inspired generations of researchers in the pursuit of unveiling a variety of sensory capacities in the animal world (Hölldobler and Lindauer 1985; Dyer and Arikawa 2014). The subsequent use of transdisciplinary research approaches to understand sensory perception also resulted in insights into how physics principles are used by biological organisms to enable solutions to complex problems (Hölldobler and Lindauer 1985; Barth et al. 2012; Galizia et al. 2012). In early 1949, whilst still at the University of Graz in Austria before returning to Munich in Germany, von Frisch had published one of his most important findings that honeybees could communicate the location of rewarding flowers with conspecifics via a symbolic dance language (von Frisch 1949). The bee dance language represents the vector direction and distance of a patch of flowers from the hive, and uses as a key reference the position of the sun or the inferred position via the degree of polarization of the sky if the sun is obscured by clouds. The discovery of the symbolic bee dance language by von Frisch generated immense interest amongst biologists, and Professor WH Thorpe of Cambridge University in England had visited von Frisch in Austria during September 1948 and repeated the experiments, which were published (Thorpe 1949) as a confirmation study in Nature on the 2nd of July 1949. Years later in 1973, together with Konrad Lorenz and Nikolaas Tinbergen, von Frisch was awarded the Nobel Prize in Physiology or Medicine. In the written acceptance speech for receiving the Nobel Prize, which was delivered by his son Otto von Frisch due to poor health of Karl von Frisch at the time, he prominently discusses the importance of the seminal bee dance language studies published in 1949 (von Frisch 1973).

## Einstein meets von Frisch at Princeton University

In April 1949, von Frisch was invited to present a lecture at Princeton University in the United States of America. It is known that Einstein attended the lecture by von Frisch, and von Frisch himself seems to have been impressed as he writes in his memoir (von Frisch 1962 p145: translation Natalie Barth): “*New York was followed by a visit to Princeton. During my lecture there the striking head of ALBERT EINSTEIN captivated me among the audience. Outstandingly intelligent faces in the audience are useful, I find, for the lecturer. One makes an extra effort to pull oneself together. On the other hand, at popular lectures, I also observe with pleasure a particularly witless countenance. If a glimmer of understanding appears on such a face, I know that I am on the right path. EINSTEIN invited us to visit him in his laboratory, where, the next day, we engaged in a friendly dispute with this humorous man. It was more than 40 years ago that my physicist uncle FRANZ EXNER had tried to convey to us clueless laymen in a small circle of family and friends an idea of EINSTEIN's ingenious and at that time still new achievement.*” It is not known exactly what else the two professors discussed in their meeting at Princeton University in April 1949, but a previously unknown letter written by Einstein to Glyn Davys on the 18th October 1949 permits some new insights into the meeting of von Frisch and Einstein.

## Glyn Davys contacts Einstein

Michael Normal “Glyn” Davys was born in 1925, and in 1942 joined the British Royal Navy where he was trained as an engineer. In 1946, he was transferred to the aircraft carrier HMS Illustrious to do research on RADAR where he served until September 1947. He then went to the Central School of Drama to study acting, before working as an actor, producer and director in the theatre and television industry during the 1950s. It appears that Glyn Davys had written to Einstein at Princeton University or the Institute for Advanced Study in Princeton with a query about animal perception and physics. However, after recently searching known repositories of Einstein’s records, we have not yet been able to locate the original letter written by Glyn Davys and answered by Einstein. Although the letter may no longer exist, by tracing available evidence, it is possible to infer when the letter was written, and the likely topic of the letter.

## Skylight polarization and Einstein’s response to Davys

With the publication of the findings on the effects of the polarization pattern of the sky on the dances and the orientation of honeybees (von Frisch 1949; Thorpe 1949), there was almost immediate media and public interest in England. In his paper, Thorpe (1949) writes “*while the mechanism of perception of the polarisation remains obscure, the essential facts seem to be well established, and it appears that we are justified in the conclusion which von Frisch himself reaches, that the polarisation of the light of the sky is an effective indication for bee orientation*”. This publication in Nature enabled public access to the findings of von Frisch and on the 7th of July 1949 The Guardian newspaper in London disseminated how the findings about the bee dance language were enabled by polarization sensitive vision. This article, or secondary reporting of the news in England, is thus the most likely source for how Glyn Davys became aware of the new research findings of von Frisch on bee sensory perception. These dates in 1949 fit with the time frame for Professor Einstein to receive a letter from Glyn Davys, and then write a reply letter dated 18th October 1949. Glyn Davys passed away on the 4th January 2011 (Association pour la Promotion des Extraits Foliaires en Nutrition 2012). However, recent correspondence with his son John Davys in 2021 confirms that he recalls as an adult a conversation with his father who said that he “*was interested in the bees’ use of the plane of polarisation of sunlight as a navigational aid*”. It is thus likely that this is at least one topic of the letter that Glyn Davys wrote to Professor Einstein as he had access to publicly available and relevant information at the correct timeframe, and communicated an interest in that topic. The return letter from Einstein suggests Glyn Davys must have specifically mentioned bees and von Frisch since this is the topic of the response letter. Having worked on RADAR research in 1946 and 1947, Glyn Davys had most likely also been well primed to thinking about potential relationships between physics and biology. In the 1940s, physics principles had been used to develop RADAR to enable remote monitoring of aircraft and ships (Watson Jr 2009). However, it would have been common for most people in the 1940s to have assumed with a strong anthropocentric bias that animals might only employ the traditional ‘five human senses’ (sight, sound, touch, smell, and taste) to interact with their environment. Interestingly, as a remarkable coincidence to the important physics and engineering breakthroughs in RADAR during the 1940s, the independent investigation of echolocation or “bio-radar” by bats was published around the same time (Griffin 1944; Grinnell 2018). This coincidence of science started to increase attention to what sensory capabilities different animals may have, and whether these could be studied to understand the physics principles required for improving and designing innovative solutions of technical problems. Glyn Davys writing to Einstein in 1949 was thus very likely forward thinking about future possibilities regarding navigational technologies.

**Fig. 2:**
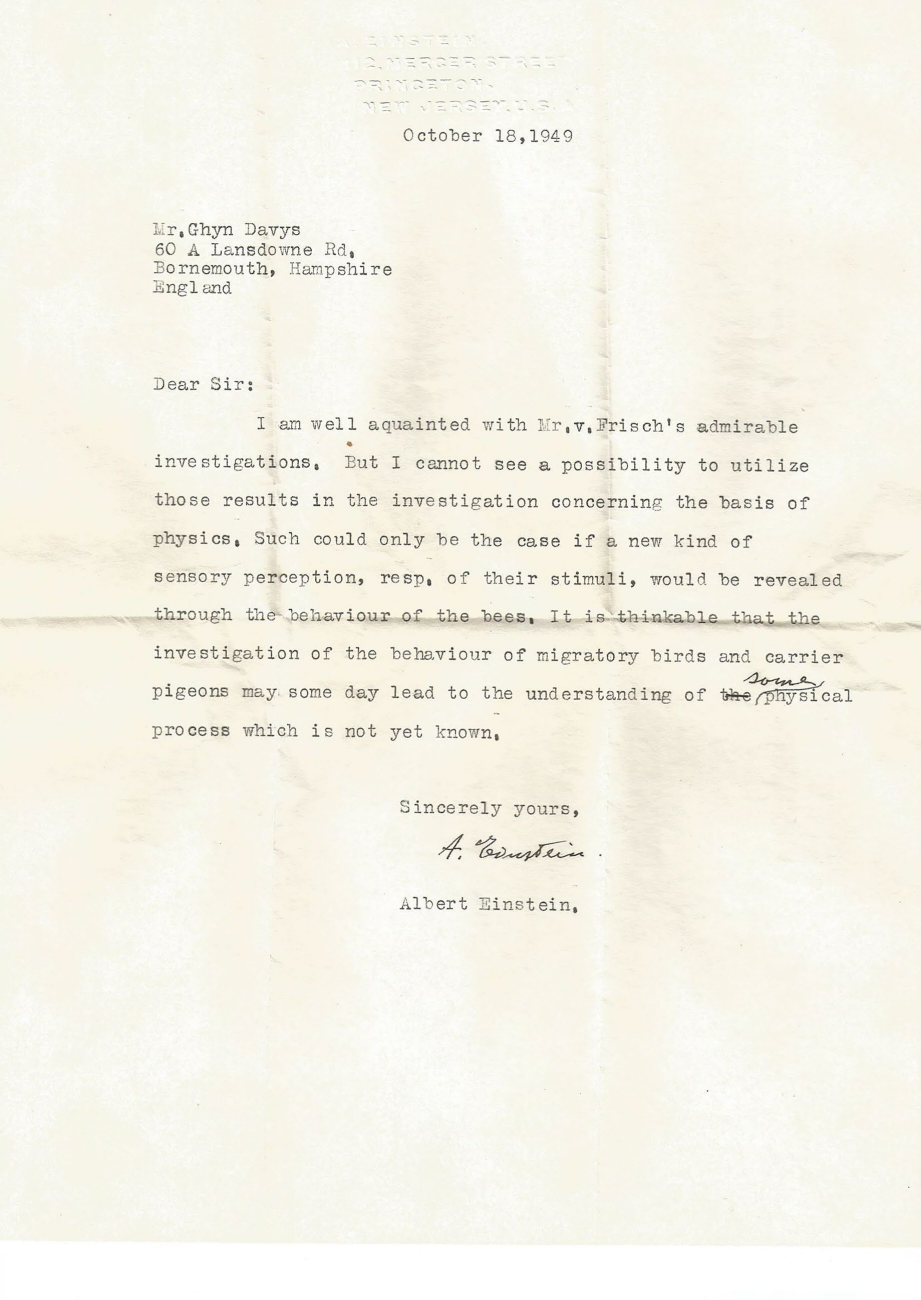
Letter dated 18th October 1949 by Professor Albert Einstein from Princeton (USA) to Mr Glyn (written Mr Ghyn [sic]) Davys in England with reference to the work of von Frisch and sensory perception of animals

The response letter written by Einstein is reproduced here for the first time (Fig. 2) with permission of the Davys family which has had sole possession of the letter since 1949. It is almost certain that until now the content of the letter has remained unknown to the scientific community and the general public. Glyn Davys mentioned the existence of the letter to his children when they were young, but he himself thought that the letter was lost following several moves during his life. It only came to light when his wife, Judith Davys, was sorting out his papers after his death in 2011 and subsequently put the Einstein letter away for safe keeping. A copy of the letter was recently provided to The Albert Einstein Archives at The Hebrew University of Jerusalem, where Professor Einstein bequeathed his notes, letters and records, and the letter was authenticated as genuine. The letter is also briefly mentioned in a newsletter referring to the life of Glyn Davys (Association pour la Promotion des Extraits Foliaires en Nutrition 2012).

Whilst the typed letter by Einstein reproduced in Fig. 2 is brief, it does contain some interesting thoughts about our present knowledge of animal sensory perception. From the first sentence of the letter “*I am well acquainted with Mr. v. Frisch’s admirable investigation*” it is clear that von Frisch’s ideas about bee sensory perception had remained present in Einstein’s thinking since his meeting at Princeton University with von Frisch six months earlier.

In his letter (Fig. 2) Einstein continues: “*But I cannot see a possibility to utilize those results in the investigation concerning the basis of physics. Such could only be the case if a new kind of sensory perception, resp. of their stimuli, would be revealed through the behaviour of the bees*”. Einstein thus appeared open to the possibilities of undiscovered sensory cues, and how that may open our eyes and minds to new possibilities and technological developments.

Although Einstein could not have known it, in more recent times, the behaviour of bees has revealed some novel and interesting phenomena about how the world can be sensed in alternative ways. These discoveries have already led to improvements in various technologies like that of sensors, robotics, and artificial intelligence (Barth et al. 2012; Srinivasan et al. 2012; Srinivasan 2020).

A very complex problem many animals must solve is to know their own location in an environment as they travel in search of food, mates and/or shelter, and there are multiple examples of sophisticated navigational abilities in different insect models. In a two-dimensional environment for terrestrial animals like ants, this is already a complex problem for which the solution requires the integration of multiple cues (Wystrach et al. 2014; Wehner 2020). For example, the African ball-rolling dung beetle can navigate using the sun, the moon, the celestial polarization pattern, stars, and the Milky Way (Dacke et al. 2013). Flying bees, birds or unmanned flying machines navigate in a three-dimensional environment where the dimension of height adds enormously to the computational requirements on a brain to reliably map spatial location (Srinivasan 2020).

Classically, it was proposed that bees might have a mechanism to gauge distance via energy consumption (Heran and Wanke 1952; von Frisch 1967). However, subsequent research has shown that honeybees actually employ a visually driven odometer that detects the amount of optic flow to judge distance (Esch et al. 2001; Srinivasan 2000; Srinivasan et al. 2012). Such a robust mechanism can be translated into machine coding to enable unmanned aeronautical vehicles to autonomously operate in complex environments (Srinivasan 2011; Srinivasan et al. 2012). Other recent research has revealed that bumblebees can detect and behaviourally use weak electric fields to economize their foraging (Clarke et al. 2013), and honeybees have a capacity to detect the Earth’s magnetic field potentially either by signals transmitted from the short wavelength photoreceptor, as reported for *Drosophila* (Gegear et al. 2008), and/or by ferromagnetic crystals present in the bee’s abdomen (Liang et al. 2016)***.*** Thus, 70 years after Einstein thought and typed his ideas about “*Such could only be the case if a new kind of sensory perception, resp.* [respectively] *of their stimuli, would be revealed through the behaviour of bees*”, there are new findings about the behaviour of bees and other animals. Novel sensory and perceptual capabilities are indeed still being discovered and providing bio-inspired ideas employing physics and engineering principles for the development of solutions to complex problems. However, whilst such recent discoveries do bridge our knowledge between separate fields, as Einstein writes, such work might not be informing the basis of physics. For example, Einstein had seen in April 1949 von Frisch’s work involving polarization perception in bees which was a beautiful example of how biology can utilise physics principles, but in itself was not at the forefront of physics innovation.

## Novel insights in long-distance navigation

Consider the second of Einstein’s thoughts in the letter (Fig. 2), “*It is thinkable that the investigation of the behaviour of migratory birds and carrier pigeons may someday lead to the understanding of some physical process which is not yet known”.* It is amazing that he conceived this possibility, decades before empirical evidence revealed that several animals can indeed perceive magnetic fields and use such information for navigation (Walker et al. 2002). Considering homing pigeons, behavioural studies show that individual birds with occluded vision that have travelled a long distance can orientate to within 2 km of their loft by sensing magnetic fields, although without vision they cannot precisely locate it (Schmidt-Koenig and Walcott 1978; Gould 1982). The navigation of migratory birds reveals the potential use of multiple sensory cues like stars, sun, geomagnetic field, and polarized light for orientation (Beason and Wiltschko 2015). Research on *Catharus* thrushes fitted with radio transmitters shows that these birds use a magnetic compass as the primary orientation guide in flight. The magnetic compass is calibrated daily relative to the solar azimuth during the sunset and/or twilight period to compensate for angle change relative to current position. Due to the large distances that many birds fly to return to a specific breeding site every year, this is a task that requires the precise calculation of the destination with little margin for error (Cochran et al. 2004).

Despite much recent research on the biophysics principles enabling such long-distance navigation, there is still considerable debate over the exact mechanism allowing for such a feat. Nevertheless, radical formation by cryptochrome proteins in avian retinas are potentially an important component of the ability to sense magnetic fields in various vertebrate and invertebrate species (Ritz et al. 2002; Hiscock et al. 2016), but the precise physics principles underpinning such an animal perception is still a topic of intense investigation. Perhaps ironically, it is also conjectured that the radical pair mechanism is an example of non-trivial quantum biology operating at the nano- and subnanometer scale, which explicitly utilizes quantum randomness, superposition and even quantum entanglement (Ritz et al. 2004; Hore and Mouritsen 2016; Marais et al. 2018). The irony is that although Einstein (along with Max Planck) introduced science to quantum mechanics, he famously rejected quantum randomness and the entanglement as ‘spooky action at a distance’ (Letter from Einstein to Max Born, 3 March 1947; Born et al. 1971). However, the openness of Einstein’s mind to novel possibilities observed in nature is clearly shown in the letter to Glyn Davys (Fig. 2), and over 70 years later possibilities envisaged by Einstein do remain an open field of active research.

The impressive use of multisensory information by animals to make complex and behaviourally relevant decisions requires demanding and energy efficient processing. The bar-tailed godwit (*Limosa lapponica baueri*) has been satellite-tracked flying nonstop 11,000-km from Alaska to New Zealand (Gill et al. 2009), a navigation task that must have a very slim budget for information processing. Honeybees also navigate in complex 3D environments on very tight energy budgets (von Frisch 1967; Srinivasan 2011). They extract statistical information about co-occurrence contingencies of visual scenes (Avarguès-Weber et al. 2020), suggesting that the way tiny insect brains enable such tasks still contains many lessons for improving computer and robot design (Srinivasan 2020). The study of insects which can navigate by polarized skylight such as crickets, locusts, flies, bees, ants, and dung beetles has resulted in bio-inspired polarized skylight-based navigation sensors. Such devices include the (1) polarization navigation sensor on photodetector with linear film polarizers, (2) camera-based polarization sensors, and (3) division of focal plane (DOFP) polarimeter-based complementary metal–oxide–semiconductor (CMOS) polarization imaging sensors. Any of these sensors could potentially be used to develop a miniaturized bio-inspired navigation device for humans (Karman et al. 2012).

Scientists like Einstein have an amazing effect on our physical and intellectual existence, often well beyond their own field and time (Hawking 2002). Glyn Davys was grateful to have received a reply from Einstein to his letter (Association pour la Promotion des Extraits Foliaires en Nutrition 2012), and went on to publish some of his own research (Vyas et al. 2010).

## Advanced understanding of bee cognition underlines Einstein’s foresight

The honeybee has become an important model in research aiming at an understanding of how a miniature brain can process information (Giurfa 2007, 2019; Srinivasan 2020). At the same time, there has been considerable interest in potentially bio-inspired solutions for the design of efficient computers (Merolla et al. 2014) with reduced energy consumption (Masanet et al. 2020). Bees have demonstrated their capacity to perform many cognitive-like tasks including maze navigation (Zhang et al. 2000), face processing (Avarguès-Weber et al. 2018) and multimodal processing (Giurfa et al. 2001; Ravi et al. 2016; Solvi et al. 2020), analogous to what large brained primates can achieve (Chittka and Niven 2009; Avarguès-Weber et al. 2020). The publication of two recent studies showing that bees can learn to understand the mathematical concept of zero (Howard et al. 2018) and also perform basic arithmetic (Giurfa 2019; Howard et al. 2019) received wide media coverage around the world (Dyer et al. 2020). Judith Davys, the wife of the late Glyn Davys, heard a BBC radio coverage about what bees can do, including these two studies, and recalled the letter written to her husband by Einstein that has remained all this time in the possession of the family. She contacted one of us (AGD) and provided a copy of the letter shown in Fig. 2.

All the discoveries mentioned by us, and many other related scientific breakthroughs since Einstein’s letter of 1949, promise that there is still much to be learnt from multidisciplinary studies incorporating general biology, behavioural ecology, neuroethology, physics and engineering (Barth et al. 2012). For example, the presence of multiple photoreceptors in a single polychromatic rhabdom of a butterfly’s or many other insects’ ommatidium allows the resolution of different wavelengths in a single pixel at the limit of spatial sensitivity (Takeuchi et al. 2006). This design principle can work for one-shot multilayer array sensors for digital cameras which can differentially absorb photons depending on the wavelength of light and enable multispectral sensing (Lyon and Hubel 2002). A more recent innovation comes from the complex visual system of the mantis shrimp (*Neogonodactylus oerstedii*) (Marshall and Oberwinkler 1999; Thoen et al. 2014; Marshall and Arikawa 2014) and the design of a hyperspectral photo detector mimicking the spectral and polarimetric sensing capabilities of mantis shrimps (Altaqui et al. 2021). Another such case is our knowledge of the dolphin sonar, which enabled engineers to improve shallow water sonar using bio-inspired physics principles (Au 2004; Dobbins 2007). Finally, the studies of bee vision led to efficient new solutions of colour mapping for machine vision (Garcia et al. 2017). Thus, whilst no further record of the meeting between Einstein and von Frisch is known to exist, the glimpses at the short letter from Einstein to Glyn Davys suggest that the two researchers most likely discussed how knowledge of animal sensory perception can inspire new discoveries by exploring mechanistic explanations of how animals operate in complex environments. They very likely would have been impressed by what modern science has revealed about their respective and sometimes overlapping fields of interest by asking nature questions with an emphasis on theory and experiment, respectively. If further information about the assumed lost letter from Glyn Davys to Einstein does still exist, it would be of value to consider it in relation to the current manuscript, and how innovative researchers like Einstein and von Frisch engaged the thinking of people at their time and beyond.
