# Correction to: Einstein, von Frisch and the honeybee: a historical letter comes to light

**DOI:** 10.1007/s00359-021-01497-z

**Published:** 2021-06-21

**Authors:** Adrian G. Dyer, Andrew D. Greentree, Jair E. Garcia, Elinya L. Dyer, Scarlett R. Howard, Friedrich G. Barth

**Affiliations:** 1 School of Media and Communication, RMIT University, Melbourne, VIC 3001 Australia; 2 Department of Physiology, Monash University, Clayton, VIC 3800 Australia; 3 ARC Centre of Excellence for Nanoscale BioPhotonics, School of Science, RMIT University, Melbourne, VIC 3001 Australia; 4 Department of Computer Science and Software Engineering, Swinburne University of Technology, Hawthorn, VIC 3122 Australia; 5 Centre for Integrative Ecology, School of Life and Environmental Sciences, Deakin University, Burwood, VIC 3217 Australia; 6 Department of Neurosciences and Developmental Biology, Faculty of Life Sciences, University of Vienna, Althanstr.14, 1090 Vienna, Austria

## Correction to: Journal of Comparative Physiology A 10.1007/s00359-021-01490-6

Authors would like to update few corrections to their publication

Corrections to the list of references updated

Minor text corrections updated

The original article has been corrected.

